# Repair of rotator cuff tears in patients aged 75 years and older: Does it make sense? A systematic review

**DOI:** 10.3389/fpubh.2022.1060700

**Published:** 2023-01-17

**Authors:** Chenyang Meng, Boyong Jiang, Ming Liu, Fujia Kang, Lingyue Kong, Ting Zhang, Caixia Wang, Jingjuan Wang, Changxu Han, Yizhong Ren

**Affiliations:** ^1^The Second Affiliated Hospital of Inner Mongolia Medical University, Hohhot, Inner Mongolia Autonomous Region, China; ^2^Inner Mongolia Medical University, Hohhot, Inner Mongolia Autonomous Region, China

**Keywords:** rotator cuff tear, arthroscopic repair, open repair, elderly, shoulder

## Abstract

**Background:**

Rotator cuff injuries are common, and morbidity increases with age. The asymptomatic full-thickness tear rate is 40% in the over 75-year-old population.

**Purpose:**

This study aimed to systematically review the literature on the outcomes of rotator cuff repair among >75 years old patients.

**Study design:**

Systematic review.

**Methods:**

A systematic review of the literature was performed following the PRISMA (Preferred Reporting Items for Systematic Reviews and Meta-Analyses) guidelines. A literature search was performed in the electronic databases of PubMed, Medline, Embase, and The Cochrane Library. Studies in English evaluating repair of full-thickness rotator cuff tears in patients aged >75 years were included.

**Results:**

Six studies were reviewed, including 311 patients (313 shoulders) treated with arthroscopic and/or open rotator cuff repair. Sixty-one patients were lost to follow-up, leaving 252 shoulders with outcome data. Patients in this age group demonstrated a significant improvement in the clinical and functional scores after rotator cuff repair, with a high satisfaction rate. The mean American Shoulder and Elbow Surgeons scores improved from 43.8 (range, 42.0–45.5) preoperatively to 85.3 (range, 84.0 to 86.5) postoperatively, and the mean Constant scores improved from 45.4 (range, 34.7–55.5) to 78.6 (range, 67.0–91.6). Pain, evaluated in all studies by the visual analog scale for pain, showed a significant improvement at the last follow-up compared with the mean preoperative score. Furthermore, range of motion and return to daily activities and sports gained marked improvements.

**Conclusion:**

Rotator cuff repair in patients aged >75 years could achieve high clinical success rates with good outcomes and pain relief. Although patients in this age group are at a high risk of retear, rotator cuff repair may offer a good option with significant functional and clinical improvement.

## Introduction

Rotator cuff injuries are common, and morbidity increases with age. The rate of asymptomatic full-thickness tears is 40% in the over 75-year-old population (1, 2). Elderly patients often present various risk factors, including decreased bone quality, poor blood supply, and an increased rate of medical comorbidities. These work against the treatment of rotator cuff injuries (3, 4). Furthermore, the factors affecting tendon healing are the size and extent of the tear, presence of fatty infiltration in the rotator cuff muscle, delamination, smoking, and the rehabilitation protocol (2, 4–8). All these factors may preclude rotator cuff repair (RCR) and could make clinical outcomes less predictable.

Older individuals have a strong desire to maintain a physically active lifestyle and expect an early return to sport and occupational activities (9). These expectations typically warrant surgical management of rotator cuff tears in this population once nonoperative treatments have failed (10). Despite significant advances in arthroscopic RCR over the past 20 years, retear rates remain unacceptably high. Several studies have documented better postoperative functional gain and structural healing of the rotator cuff. Achieving good tendon healing could reasonably be considered one of the primary objectives of the surgery (11–16). Due to the large number of comorbidities observed in the elderly population and the complications and high rates of recurrent tears seen after RCR surgeries, experts have debated whether surgery was an effective treatment for rotator cuff lesions in this population. The management of rotator cuff injuries has been well studied in other age groups; however, little investigation data and no treatment guidelines are available for the over 75-year-old population.

Based on several small case series reporting successful clinical outcomes in patients older than 75 years after repair surgery (11–16), this systematic review aimed to pool these findings and evaluate the clinical outcomes of RCR in patients aged ≥75 years. In doing so, we aim to found that whether surgical treatment was beneficial for structural tendon healing with good clinical outcomes following rotator cuff injuries in this advanced age group, making it the preferred treatment option.

## Methods

This systematic review was performed following the PRISMA (Preferred Reporting Items for Systematic Reviews and Meta-Analyses) statement guidelines (17).

### Literature search strategy

Using the Cochrane Collaboration guidelines, a comprehensive search strategy was developed for the following scientific electronic databases: PubMed, and the Cochrane Library. the final search date was 31st October 2022. We used the key search terms “shoulder,” “rotator cuff,” and “repair” associated with “over” or “older,” and “75 years” to identify all related studies. The search was limited to the English language. Firstly, two independent observers were used to conduct preliminary screening of titles and abstracts, and then the full text of the selected papers was reviewed.

### Evaluation of the study quality

The methodological quality of the studies was evaluated by each author independently with the 10-item Coleman Methodology Score (CMS) (18). The CMS criteria rank articles, based on total scores, as excellent (85–100), good (70–84), fair (55–69), and poor (< 55).

### Selection criteria

We included articles reporting outcomes following arthroscopic and/or open repair of all kinds of rotator cuff tear including in patients older than 75 years. The articles had to report the outcomes of interest, including operation type, rehabilitation protocol, mean follow-up time, patient-reported outcome measures, and postoperative complications. We excluded conference abstracts, surgical techniques, reviews, clinical commentaries, and papers that were not peer-reviewed or were not written in English. No restriction was placed on sex, time since surgery, recruitment method, or rehabilitation protocol. Two reviewers independently applied the selection criteria for eligibility to articles identified during the databases search by reviewing the titles and abstract. When it was unclear whether a study was suitable for inclusion after such a review, the full text was assessed and cross-checked for eligibility. Disagreements between the reviewers were resolved by consensus, consulting a third reviewer when consensus could not be reached.

### Data extraction and synthesis

Two independent reviewers extracted the data from the included studies. Study characteristics, type of operation, clinical and radiographic follow-up intervals, patient demographics, tear size, complications, and clinical and radiographic outcomes, were extracted and documented. Retears were noted if reported based on postoperative imaging or clinical presentation following the index operation. The various clinical outcome measures included were SF-12P/M (Short Form Health Survey physical/mental components), VAS (visual analog scale), ASES (American Shoulder and Elbow Surgeons), Katz ADL (Katz Index of Independence in Activities of Daily Living), FIM motor (Functional Independence Measure motor), UCLA (University of California, Los Angeles), CS (Constant Score), SSV (Subjective Shoulder Value), SST (Simple Shoulder Test), and SANE (Single Assessment Numerical Evaluation). Subsequently, the characteristics and results of all eligible studies were synthesized. The outcomes presented inconsistent characteristics across articles, and the results were presented in a narrative description.

## Results

### Study selection

**Figure 1:**
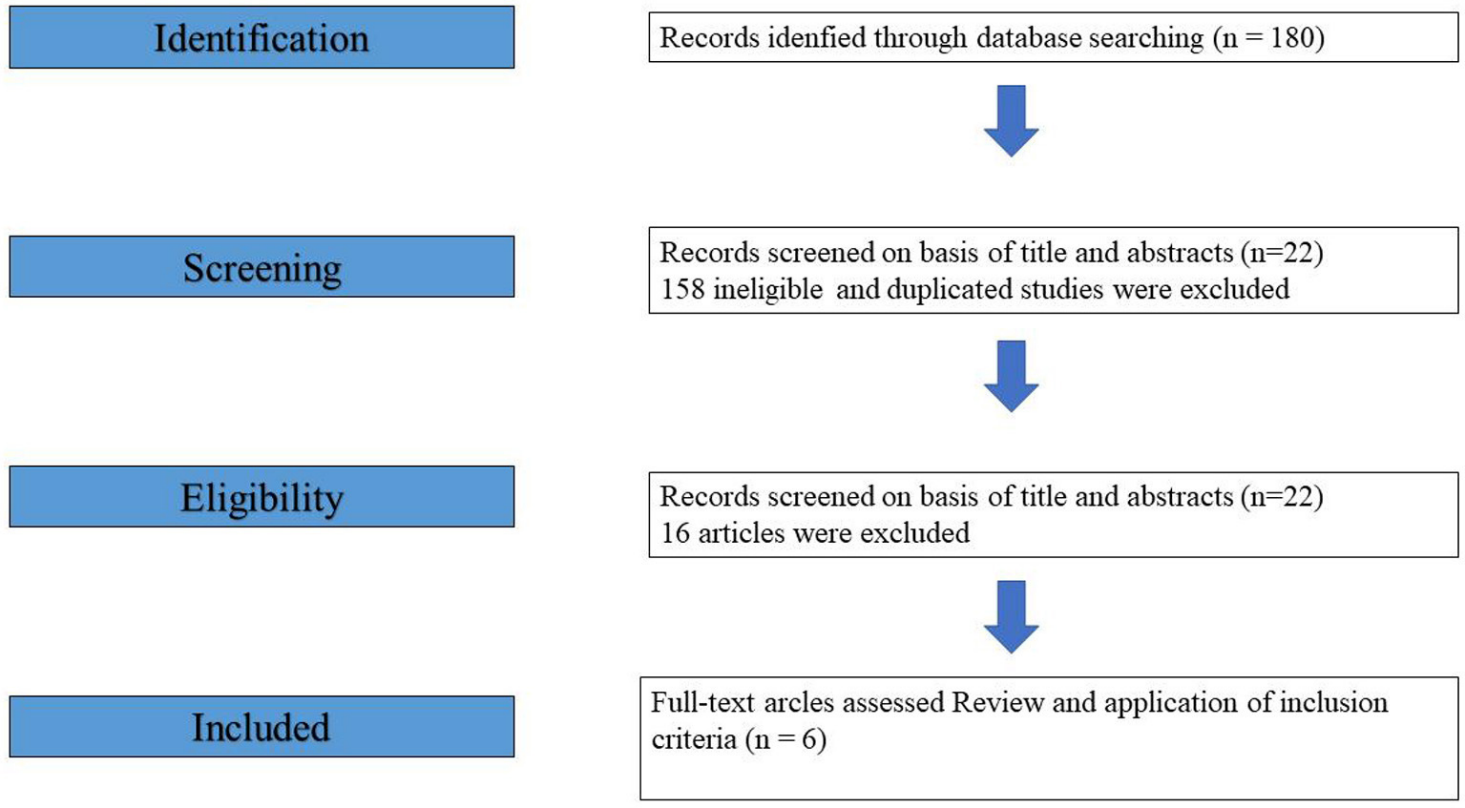
The PRISMA (Preferred Reporting Items for Systematic Meta-Analyses) flow diagram.

We initially identified 180 articles for evaluation based on the search strategy mentioned above. We excluded 158 ineligible and duplicated studies, leaving 22 articles for full-text review. Ten were excluded because they were review articles. Through a comprehensive review of the remaining articles and their citations, with a detailed search of the literature, six studies (11–16) were ultimately included in the current systematic review (Figure 1).

### Study characteristics and quality

An Excel spreadsheet (Microsoft) was developed to aggregate data from all studies. Characteristics of the included studies are shown in Table 1. The available patient cohort demographics and surgical techniques used in each study are presented in Table 2.

**Table 1:** Study characteristics.

**Study**	**Year**	**Design**	**Level of evidence**	**Mean follow-up (range), mo**	**Coleman score**
Jung et al.	2017	Retrospective	4	42(24-60)	63
Park et al.	2016	Retrospective	4	30.1 (12–108)	47
Witney-Lagen et al.	2019	Retrospective	3	26 (12-84)	72
Plachel et al.	2020	Retrospective	4	84 (36-108)	56
Stone et al.	2020	Retrospective	4	56.9(24-127)	57
Padki et al.	2021	Retrospective	3	24	71

**Table 2:** Patient demographics.

**Study**	**Shoulders (patients), *n***	**Male:female, *n***	**Age, mean ±SD (range), y**	**Follow-up, mo mean (range)**	**Operation type**	**Tear size**
Jung et al.	64 (64)	21:43	78.1 ± 4.2 (75–87)	42 (24–60)	Open (DR)	29 mass, 35 L
Park et al.	25 (25)	4:21	78.3 (75–88)	30.1 (12–108)	Arthroscopic (13 SR, 6 SB), 6 open	5 mass, 5 L, 15 S–M
Witney-Lagen et al.	60 (59)	31:28	78.4 (75–86)	26 (12–84)	Arthroscopic (32 SR, 28 DR)	25 mass, 20L, 12M, 3S
Plachel et al.	31 (30)	14:08	77 ± 2 (75–82)	84 (36–108)	Arthroscopic (10 SR, 13 DR)	6 L, 15M, 2S
Stone et al.	110 (110)	57:53	77 ± 2.6 (75–85)	56.9 (24–127)	Arthroscopic (71 SR, 24 TOE, 12 TO, 3 SubR)	22 mass, 28 L, 60 S-M
Padki et al.	23 (23)	7:16	78	24	Arthroscopic (23 DR)	NA
	313 (311)					

DR, double row; L, large; M, medium; mass, massive; NA, not available; S, small; SR, single row; TOE, transosseous equivalent repair; TO, arthroscopic transosseous repair; SB, suture bridge; SD, standard deviation; SubR, subscapularis repair.

Coleman Methodology Score was applied for quality assessment and bias analysis of the included articles, and one study was rated as poor; three studies, fair; and two studies, good. The quality score of the articles was the mean of the scores of the two investigators, 61 (range, 47–72). Articles included had evidence levels between III and IV, among which two studies (11, 14) were Level III evidence, and the remainder were Level IV evidence (Table 1).

### Patient demographics

All studies reported clinical and structural outcomes in patients aged 75 years and older. A total of 311 patients (313 shoulders) were included in the six studies, with a mean age of 77.8 (range, 77.0–78.4) years. Whether patients were recreational or professional athletes was not specified in any of the studies, and no athletic activity was reported.

### Tear type and treatment

Five studies (282 shoulders) reported the tear size: 5 small, 27 medium, 75 small / medium, 94 large, 81 massive tears, and the rest were unspecified (12–16). Four studies (11–14) used an arthroscopic approach; one study (16) used an open approach; one study (15) used both approaches. Among surgical repair techniques, 56 cases underwent single-row repair, 87 underwent double-row repair, and the remaining cases were treated by other methods. Two studies utilized double and single-row repair techniques (12, 14) two applied only the double-row repair technique (11, 12), one used the bridge technique (14), and one used various repair techniques, including single row, transosseous equivalent repair, arthroscopic transosseous repair, and isolated subscapularis repair (12).

### Patient satisfaction and functional outcomes

Studies processing the required data explored statistical significance by comparing preoperative and postoperative outcome scores (Table 3). Patient satisfaction rates were assessed in three studies (12–14). Two studies separately reported that 93.2 and 80% of the patients were satisfied with the results (13, 14). Notably, the rate of patient satisfaction in the third study was 100% (12).

**Table 3:** Outcome scores.

**Study**	**Outcome scores**	**Preoperative scores**	**Postoperative scores**	**Increase (points)**
Jung et al.	VAS, ASES, CS, Katz ADL, FIM motor (mean±SD)	6.4 ± 2.2, 42 ± 16, 44 ± 18, 3.4 ± 1.1, 25 ± 6	2.3 ± 1.1, 84 ± 8, 76 ± 7, 5.0 ± 0.8, 54 ± 7	4.1, 42, 32, 1.6, 29
Park et al.	VAS, UCLA, CS (mean±SD)	Retear group:6.0 ± 1.1, 14.4 ± 4.6, 39.5 ± 13.2/Healed group:5.2 ± 1.6, 15.8 ± 3.8, 49.3 ± 11.1	Retear group:2.4 ± 1.8, 28.3 ± 4.3, 63.6 ± 10.9/Healed group:1.4 ± 0.9, 31.1 ± 2.3, 71.9 ± 4.5	Retear group:3.6, 13.9, 24.1/Healed group:3.8, 15.3, 22.6
Witney-Lagen et al.	CS (mean±SD), Age- and sex-adjusted CS, SSV, VAS	NA, 55.5, 2.1, NA	63.4 ± 17.0, 91.6, 7.9, 2.3 ± 3.2	NA, 36.1, 5.8, NA
Plachel et al.	SST, ASES, VAS	NA	10 ± 2, 89 ± 17, 0.8 ± 2.3	NA
Stone et al.	VAS, ASES, SANE, SF-12M, SF-12P	52.5, 45.5, 35.6, 51.1, 41.6	8.7, 86.5, 83.7, 54.5, 44.3	43.8, 41.0, 48.1, 3.4, 2.7
Padki et al.	VAS, CS, UCLAS, OSS	7.0 ± 2.1, 34.7 ± 19.8, 14.3 ± 5.3, 33.9 ± 14.0	1.9 ± 2.6, 67.0 ± 8.9, 28.2 ± 5.0, 17.0 ± 7.5	5.1, 32.3, 13.9, 16.9

VAS, visual analog scale; ASES, American Shoulder and Elbow Surgeons; Katz ADL, Katz Index of Activities of Daily Living; FIM motor, functional independence measurement motor; UCLA, University of California, Los Angeles; CS, Constant score; SSV, Subjective Shoulder Value; SST, Simple Shoulder Test; SANE, Single Assessment Numerical Evaluation; SF-12P/M, Short Form Health Survey physical/mental components; OSS, Oxford Shoulder Score; NA, not available.

### Return to daily activities and sports

No study evaluated the return to sport, as no recreational or professional athletes were included in the overall patient population. Two studies (12, 13) reported outcome measures assessing the return to daily living activities. Plachel et al. (12) analyzed the activity level according to the mean SST score during daily living activities and found it significantly improved postoperatively. Jung et al. (16) found a significant improvement in the mean Katz ADL score from 3.4 to 5.0 and FIM motor score from 25 to 54, particularly in dressing, bathing, and personal hygiene.

### Range of motion

Range of motion (ROM) was analyzed in all studies except the studies by Witney-Lagen et al. (14) and Padki et al. (11). At the final follow-up, the affected shoulder showed a significant improvement in ROM, including measurements of abduction, active forward elevation, and external rotation.

### Rotator cuff integrity and retears

Rotator cuff integrity after repair was reported in 3 studies. Two studies (15, 16) performed magnetic resonance imaging (MRI) at last follow-up, and 12/46 patients (26%) (16) and 9/25 patients (36%) (15) suffered retears, respectively. In addition, the study by Stone et al. (13) relied on relevant clinical symptoms, with 5 patients out of 83 (6.0%) presenting with symptomatic retear (Table 4).

**Table 4:** Rotator cuff integrity and complications.

**Study**	**Shoulders at follow-up, *n***	**Shoulders with imaging, *n***	**Type of operation**	**Imaging**	**Retears, *n***	**Complications**
Jung et al.	64	46	Open	MRI	12	None
Park et al.	25	25	Arthroscopic	MRI	9	None
Witney-Lagen et al.	60	52	Arthroscopic	NA	NA	1 superficial infection, 1 stiffness
Plachel et al.	31	18	Arthroscopic	NA	NA	None
Stone et al.	110	83	Arthroscopic	NA	5	None
Padki et al.	23	23	Arthroscopic	NA	None	None

MRI, magnetic resonance imaging; NA, not available.

### Other complications

Other complications were reported in all studies, one (14) of which found that one patient developed a superficial portal wound infection and a stiff shoulder that were successfully resolved with antibiotics and arthroscopic capsular release, respectively. No procedure-related complications were observed in the remaining studies (Table 4).

## Discussion

With socio-economic development and improved medical conditions, the growing elderly population could now expect to live longer and more vigorously than before. Our population grows increasingly old, there is a larger proportion of patients in their 70 and 80s who remain physically active. It has been found that the rate of asymptomatic full thickness rotator cuff tears in individuals aged ≥70 years is 40% (1). Besides, it has been verified that patients younger than 60 years with rotator cuff tears rate was lower than 10% while for patients older than 80 years, the rotator cuff tear rate was 80% (19). The other studies argued that rotator cuff tears are associated with the aging process; their prevalence increases with age, reaching 80% in individuals aged over 80 years (3, 20, 21). Another study showed that the prevalence of full-thickness rotator cuff tear was at 22% for those older than 65 years, 31–41% in those older than 70, and 51% in those older than 80 (14). Since the etiology of rotator cuff injuries is likely multi-aspect and includes age-associated degeneration. The quality and function of the rotator cuff muscles seem to deteriorate with age (22–25). Based on the results of reports, it could be found that prevalence of rotator cuff tear increases with age. Combined with the epidemiology and etiology of rotator cuff injury in older people, the treatment and prognosis of rotator cuff injury in the elderly are needed to be investigated. In thus, the clinical and radiographic outcomes of RCR in elderly patients aged ≥75 years were reviewed in this study. We summarized the data from all available evidence to better understand (1) whether these older adults were suitable candidates for surgical repair of rotator cuff injuries and (2) whether surgical treatment of rotator cuff injuries in such an older cohort resulted in favorable structural and functional outcomes.

We found in this systematic review that patients older than 75 years could achieve good outcomes (11–16). Their postoperative scores were significantly higher than the preoperative scores. Furthermore, although retear of the repaired rotator cuff was reported, few complications occurred, and RCR could achieve reliable pain and function improvements in selected patients in this age group. Padki et al. performed a propensity score matched-pair analysis to compare the outcome of patients older and younger than 75 years (11). Although the 2-year postoperative OSS was 2 ± 5 points better than before surgery, the 2-year postoperative improvement in VAS, CSS, and UCLASS over the baseline was similar in both groups. Besides, the study by Witney-Lagen et al. showed that elderly patients at a mean age of 78 years benefited as much from arthroscopic RCR as their younger counterparts (mean age, 58 years) (14). Therefore, surgical treatment could be effective for older adults with symptomatic rotator cuff tears and failed conservative management, including those older than 75 years.

Older people tend to suffer from larger rotator cuff tears. Park et al. found that larger tear size was correlated with increased mean age, but the association was insignificant (15). Maman et al. reported that 54% of the symptomatic tears in patients older than 60 years increased in size while only 17% of the tears in those younger than 60 years did so (26). Besides, Gumina et al. found that patients aged >60 years were twice as likely to experience a large tear and three times more likely to experience a massive tear than younger patients (27). Although older people tend to suffer larger rotator cuff tears, clinical studies still showed significant improvements, even in patients with large to massive tears (14, 15). Tears in symptomatic older patients, especially large tears, are likely to result in tear progression and poor outcomes if they cannot be managed by surgery. Furthermore, it was reported that rotator cuff tears were a major cause of depression in the elderly population. Therefore, rotator cuff tears in the elderly, even those older than 75 years, should be treated with surgery when necessary.

It was argued that older patients have a higher risk for retear or persistent defects noted in imaging studies (28). The reported rate of retear in those aged 60 years and older was high (31–51%), with most retears occurring 3 to 6 months after repair (29, 30). However, Rhee et al. (30) reported that age was insignificantly correlated with the retear rate. Even though older patients have a higher potential for retear, the postoperative cuff integrity effect on the outcome was insignificant (31). Another report suggested that RCR significantly decreased pain and improved function and strength, despite evidence of retear on MRI (8), other studies argued that tendon nonhealing does not reflect a poor functional outcome (14, 32). While some studies showed that ROM and muscle strength were inversely correlated with a retear group (33), it has been found that physical component summaries of the UCLA and ASES socres were significantly higher in the healed group while there was no significant difference in mental component summary scores. In this systematic review, patients older than 75 years achieved good outcomes and postoperative scores higher than preoperative scores, even if retear had occurred. Although there were some reports argued that retear might affect the outcome, physical rehabilitation training after surgery might be a good choice to improve the prognosis.

Patients age and initial tear size were significantly associated with the severity of osteoarthritic changes through a long-term follow-up (34). Plachel et al. found that significant progression of secondary glenohumeral osteoarthritis occurred in patients older than 75 years (12). However, they thought that this progression was the natural course of the disease during that period of one's life rather than secondary to a persistent rotator cuff lesion since similar progression occurred in the non-affected shoulder.

The most important result was that patients older than 75 years at the time of surgery achieved good clinical results and reported high patient satisfaction at midterm follow-up. Therefore, RCR offers a joint-preserving option with significant functional and clinical improvement for patients older than 75 years without advanced muscle degeneration. However, it should be noted that massive tears had a higher risk for subsequent reverse total shoulder arthroplasty.

Several limitations remain in the present study. First, heterogeneity should not be underestimated. The interventions and objectives of the included studies were similar, but they differed in population characteristics, tear sizes, repair type (i.e., open or / and arthroscopic), repair construct (i.e., single-row, transosseous equivalent double-row, or arthroscopic transosseous tunnel repair), and outcome scores. Second, due to the limited number of included studies and overall low level of evidence (3 and 4), more case-control design studies are needed to provide better evidence. Furthermore, these studies have different follow-up intervals, which might disturb the observation of effects on function.

## Conclusion

RCR could achieve high clinical success rates with good outcomes and pain relief in patients aged over >75 years. Although patients in this age group are susceptible to retears, RCR may offer a good option with significant functional and clinical improvements.

## Data availability statement

The original contributions presented in the study are included in the article/supplementary material, further inquiries can be directed to the corresponding authors.

## Author contributions

CH and YR were in charge of the conceptualization, supervision, and project administration. CM, BJ and ML designed and performed the study and were the main writers of the manuscript. FK, LK, CW, TZ, and JW fulfilled validation and formal analysis. All authors read and approved the final manuscript.

## References

[1] Kim HM Teefey SA Zelig A Galatz LM Keener JD Yamaguchi K. Shoulder strength in asymptomatic individuals with intact compared with torn rotator cuffs. J Bone Joint Surg Am. (2009) 91:289–96. 10.2106/JBJS.H.00219 19181972 PMC2663343

[2] Milgrom C Schaffler M Gilbert S van Holsbeeck M. Rotator-cuff changes in asymptomatic adults. The effect of age, hand dominance and gender. J Bone Joint Surg Br Vol. (1995) 77:296–8. 10.1302/0301-620X.77B2.7706351 7706351

[3] Brewer BJ. Aging of the rotator cuff. Am J Sports Med. (1979) 7:102–10. 10.1177/036354657900700206 434288

[4] Chung SW Oh JH Gong HS Kim JY Kim SH. Factors affecting rotator cuff healing after arthroscopic repair: osteoporosis as one of the independent risk factors. Am J Sports Med. (2011) 39:2099–107. 10.1177/0363546511415659 21813440

[5] Gallagher BP Bishop ME Tjoumakaris FP Freedman KB. Early versus delayed rehabilitation following arthroscopic rotator cuff repair: a systematic review. Phys Sportsmed. (2015) 43:178–87. 10.1080/00913847.2015.1025683 25797067

[6] O'Donnell EA Fu MC White AE Taylor SA Dines JS Dines DM . The effect of patient characteristics and comorbidities on the rate of revision rotator cuff repair. Arthroscopy. (2020) 36:2380–8. 10.1016/j.arthro.2020.05.022 32654928

[7] Santiago-Torres J Flanigan DC Butler RB Bishop JY. The effect of smoking on rotator cuff and glenoid labrum surgery: a systematic review. Am J Sports Med. (2015) 43:745–51. 10.1177/0363546514533776 24859982

[8] Cho NS Rhee YG. The factors affecting the clinical outcome and integrity of arthroscopically repaired rotator cuff tears of the shoulder. Clin Orthop Surg. (2009) 1:96–104. 10.4055/cios.2009.1.2.96 19885061 PMC2766755

[9] Bhatia S Greenspoon JA Horan MP Warth RJ Millett PJ. Two-year outcomes after arthroscopic rotator cuff repair in recreational athletes older than 70 years. Am J Sports Med. (2015) 43:1737–42. 10.1177/0363546515577623 25834140

[10] Hantes ME Ono Y Raoulis VA Doxariotis N Venouziou A Zibis A . Arthroscopic single-row versus double-row suture bridge technique for rotator cuff tears in patients younger than 55 years: a prospective comparative study. Am J Sports Med. (2018) 46:116–21. 10.1177/0363546517728718 28942685

[11] Padki A Chen JY Lee MJH Ang BFH Lie DTT. Septuagenarians aged 75 years and older do benefit from arthroscopic rotator cuff repair: a propensity matched-pair analysis. JSES Int. (2021) 5:459–62. 10.1016/j.jseint.2020.12.020 34136854 PMC8178637

[12] Plachel F Siegert P Rüttershoff K Akgün D Scheibel MJJoS Surgery E. Clinical midterm results of arthroscopic rotator cuff repair in patients older than 75 years. (2020) 29:1815–20. 10.1016/j.jse.2020.01.093 32146044

[13] Stone MA Ho JC Kane L Lazarus M Namdari SJJoS Surgery E. Midterm outcomes of arthroscopic rotator cuff repair in patients aged 75 years and older. (2020) 29:S17–22. 10.1016/j.jse.2019.11.022 32088076

[14] Witney-Lagen C Mazis G Bruguera J Atoun E Sforza G Levy O. Do elderly patients gain as much benefit from arthroscopic rotator cuff repair as their younger peers? J Shoulder Elbow Surg. (2019) 28:1056–65. 10.1016/j.jse.2018.10.010 30704915

[15] Park JG Cho NS Song JH Baek JH Jeong HY Rhee YG. Rotator cuff repair in patients over 75 years of age: clinical outcome and repair integrity. Clin Orthop Surg. (2016) 8:420–7. 10.4055/cios.2016.8.4.420 27904725 PMC5114255

[16] Hong JJ Sim GB Bae KH Kekatpure AL Jeon IHJJoS Surgery E. Rotator cuff surgery in patients older than 75 years with large and massive tears. (2017) 26:265–72. 10.1016/j.jse.2016.07.004 27720414

[17] Page MJ McKenzie JE Bossuyt PM Boutron I Hoffmann TC Mulrow CD . The PRISMA 2020 statement: An updated guideline for reporting systematic reviews. Int J Surg. (2021) 88:105906. 10.1016/j.ijsu.2021.105906 33789826

[18] Coleman BD Khan KM Maffulli N Cook JL Wark JD. Studies of surgical outcome after patellar tendinopathy: clinical significance of methodological deficiencies and guidelines for future studies. Victorian Institute of Sport Tendon Study Group. Scand J Med Sci Sports. (2000) 10:2–11. 10.1034/j.1600-0838.2000.010001002.x 10693606

[19] Minagawa H Yamamoto N Abe H Fukuda M Seki N Kikuchi K . Prevalence of symptomatic and asymptomatic rotator cuff tears in the general population: From mass-screening in one village. J Orthop. (2013) 10:8–12. 10.1016/j.jor.2013.01.008 24403741 PMC3768248

[20] Tempelhof S Rupp S Seil R. Age-related prevalence of rotator cuff tears in asymptomatic shoulders. J Shoulder Elbow Surg. (1999) 8:296–9. 10.1016/S1058-2746(99)90148-9 10471998

[21] Hattrup SJ. Rotator cuff repair: relevance of patient age. J Shoulder Elbow Surg. (1995) 4:95–100. 10.1016/S1058-2746(05)80061-8 7600171

[22] Romeo AA Hang DW Bach BR Jr. Shott S. Repair of full thickness rotator cuff tears. Gender, age, and other factors affecting outcome. Clin Orthop Relat Res. (1999) 367:243–55. 10.1097/00003086-199910000-00031 10546622

[23] Gladstone JN Bishop JY Lo IK Flatow EL. Fatty infiltration and atrophy of the rotator cuff do not improve after rotator cuff repair and correlate with poor functional outcome. Am J Sports Med. (2007) 35:719–28. 10.1177/0363546506297539 17337727

[24] Goutallier D Postel JM Bernageau J Lavau L Voisin MC. Fatty muscle degeneration in cuff ruptures. Pre- and postoperative evaluation by CT scan. Clin Orthop Relat Res. (1994) 304:78–83. 10.1097/00003086-199407000-00014 8020238

[25] Cofield RH Parvizi J Hoffmeyer PJ Lanzer WL Ilstrup DM Rowland CM. Surgical repair of chronic rotator cuff tears. A prospective long-term study. J Bone Joint Surg Am. (2001) 83:71–7. 10.2106/00004623-200101000-00010 11205861

[26] Maman E Harris C White L Tomlinson G Shashank M Boynton E. Outcome of nonoperative treatment of symptomatic rotator cuff tears monitored by magnetic resonance imaging. J Bone Joint Surg Am Vol. (2009) 91:1898–906. 10.2106/JBJS.G.01335 19651947

[27] Gumina S Carbone S Campagna V Candela V Sacchetti FM Giannicola G. The impact of aging on rotator cuff tear size. Musculoskelet Surg. (2013) 97 Suppl 1:69–72. 10.1007/s12306-013-0263-2 23588834

[28] Altintas B Anderson NL Pitta R Buckley PS Bhatia S Provencher MT . Repair of rotator cuff tears in the elderly: does it make sense? A Systematic Review. Am J Sports Med. (2020) 48:744–53. 10.1177/0363546519834574 31038992

[29] Bishop J Klepps S Lo IK Bird J Gladstone JN. Flatow ELJJoS . Cuff integrity after arthroscopic versus open rotator cuff repair: a prospective study. J Shoulder Elbow Surg. (2006) 15:290–9. 10.1016/j.jse.2005.09.017 16679227

[30] Rhee YG Cho NS Yoo JH Surgery R. Clinical outcome and repair integrity after rotator cuff repair in patients older than 70 years versus patients younger than 70 years. Arthroscopy. (2014) 30:546–54. 10.1016/j.arthro.2014.02.006 24630958

[31] Klepps Medicine S. Prospective evaluation of the effect of rotator cuff integrity on the outcome of open rotator cuff repairs. Am J Sports Med. (2004) 32:1716. 10.1177/0363546504265262 15494338

[32] Liu SH Baker CL. Arthroscopically assisted rotator cuff repair: correlation of functional results with integrity of the cuff. Arthroscopy. (1994) 10:54–60. 10.1016/S0749-8063(05)80293-2 8166903

[33] Cole BJ McCarty LP. 3rd Kang RW Alford W Lewis PB Hayden JK. Arthroscopic rotator cuff repair: prospective functional outcome and repair integrity at minimum 2-year follow-up. J Shoulder Elbow Surg. (2007) 16:579–85. 10.1016/j.jse.2006.12.011 17629505

[34] Flurin PH Hardy P Valenti P Meyer N Kempf JFJRdCOeT. Osteoarthritis after rotator cuff repair: a 10-year follow-up study. Orthop Traumatol Surg Res. (2016) 103:477–81. 10.1016/j.otsr.2017.03.007 28347783

